# Measurement of Flow Volume in the Presence of Reverse Flow with Ultrasound Speckle Decorrelation

**DOI:** 10.1016/j.ultrasmedbio.2019.07.001

**Published:** 2019-11

**Authors:** Xiaowei Zhou, Xinhuan Zhou, Chee Hau Leow, Meng-Xing Tang

**Affiliations:** Department of Bioengineering, Imperial College London, London, United Kingdom

**Keywords:** Speckle decorrelation, Volumetric flow rate, Microbubbles, 3-D flow, Reverse flow, Ultrasound imaging velocimetry

## Abstract

Direct measurement of volumetric flow rate in the cardiovascular system with ultrasound is valuable but has been a challenge because most current 2-D flow imaging techniques are only able to estimate the flow velocity in the scanning plane (in-plane). Our recent study demonstrated that high frame rate contrast ultrasound and speckle decorrelation (SDC) can be used to accurately measure the speed of flow going through the scanning plane (through-plane). The volumetric flow could then be calculated by integrating over the luminal area, when the blood vessel was scanned from the transverse view. However, a key disadvantage of this SDC method is that it cannot distinguish the direction of the through-plane flow, which limited its applications to blood vessels with unidirectional flow. Physiologic flow in the cardiovascular system could be bidirectional due to its pulsatility, geometric features, or under pathologic situations. In this study, we proposed a method to distinguish the through-plane flow direction by inspecting the flow within the scanning plane from a tilted transverse view. This method was tested on computer simulations and experimental flow phantoms. It was found that the proposed method could detect flow direction and improved the estimation of the flow volume, reducing the overestimation from over 100% to less than 15% when there was flow reversal. This method showed significant improvement over the current SDC method in volume flow estimation and can be applied to a wider range of clinical applications where bidirectional flow exists.

## Introduction

The volume of blood flowing into a specific organ or tissue is the most relevant factor determining whether the organ can get sufficient oxygen and nutrients for its metabolic demand. The supply of blood through blood vessels can be impaired by cardiovascular diseases. Accurately measuring the volumetric flow rate in blood vessels could be potentially useful in a variety of clinical applications, such as assessing the cardiac output (Allsager and Swanevelder, 2003, Mehta, 2014), determining the degree of vessel stenosis in coronary arteries (Petretta et al. 2011), monitoring the blood supply to the brain (Likittanasombut et al. 2006), evaluating kidney or liver failure and measuring the effects of the corresponding pharmacologic therapies (Burkart et al., 1993, Johnson et al., 1985, Young et al., 1996).

Direct measurement of volumetric flow rate in blood vessels remains a challenge both in clinical practice and research. Currently, magnetic resonance imaging is regarded as the gold standard (Gatehouse et al. 2005), but its application in clinics is limited by low temporal resolution and poor accessibility. Ultrasound imaging is the most commonly used modality for estimating blood flow due to its affordability, real-time imaging, high temporal resolution, good and scalable spatial resolution and good accessibility. In clinical practice, although spectral Doppler and color Doppler are angle dependent, they have been used as the main ultrasound modalities for decades in investigating flow volume by multiplying the mean flow velocity with the vessel area (Gill 1985). Conventional Doppler methods have repeatedly been shown to be prone to errors from many sources, and reviews on the issues are available in the literature (Gill, 1985, Hoskins, 2010).

Instead of only detecting the flow velocity along the ultrasound beam and assuming the flow moving parallel to the vessel's long axis in the conventional Doppler methods, some advanced ultrasound techniques were proposed to have the vector flow in the scanning plane, such as vector Doppler (Dunmire et al., 2000, Yiu and Yu, 2016), 2-D particle-tracking (Poelma et al., 2009, Leow et al., 2015, Leow and Tang, 2018) and transverse oscillation (Jensen and Munk 1998). However, all these techniques can only measure the blood flow velocities within the 2-D scanning plane which means that to estimate the volumetric flow it needs to be assumed that there is axis symmetry in the velocity profile of the vessel. Picot et al. (1995) came up with an idea to estimate volumetric flow using the 2-D through-plane velocity profile obtained from the vessel's oblique transverse view with conventional color Doppler imaging. From the through-plane velocity profile, Picot's method provided a way to estimate the volumetric flow. The disadvantage of this method is that the oblique angle must be estimated when calculating the flow volume, which is not easy to obtain in practice (especially under the transverse view). A full-field view of the 3-D blood flow was reconstructed using divergence-free interpolation, but it required the vessel to be scanned at multiple locations (Zhou et al. 2019). 3-D blood flow imaging with a 2-D matrix ultrasound transducer could also be an option to solve this problem since it provides a complete estimation of flow velocities in each dimension (Holbek et al., 2017, Hudson et al., 2017). Currently, the huge amount of data and the demanding hardware and computational requirements make methods based on 2-D matrix probes difficult and costly to implement (Jensen et al. 2016).

Ultrasound speckle decorrelation (SDC) has shown the capability in estimating the through-plane flow velocity using a 1-D array transducer (Rubin et al., 2001, Park et al., 2013, Zhou et al., 2018). With the through-plane velocity, the flow volume can be calculated by integrating the velocity over the luminal area when the vessel is scanned in the transverse view. The principle of ultrasound SDC is that the SDC over time follows a specific Gaussian curve. This Gaussian-based relationship has been derived by (Wagner et al., 1983, Chen et al., 1997) and was used in a series of studies, including 3-D ultrasound imaging (Housden et al. 2007), blood flow estimations (Li et al., 1998, Rubin et al., 1999, Tuthill et al., 2012) and investigations of elastic tissue properties (Bamber and Bush, 1995, Céspedes et al., 1999). However, the application of ultrasound SDC on estimating blood flow in arteries was limited by the low frame rate of conventional ultrasound (Rubin et al., 2001, Park et al., 2013) and the weak signals coming from blood cells. The imaging frame rate must be high enough to capture the fast signal decorrelation due to rapid flow passing through the rather small elevational dimension of the transmitted acoustic beam.

In the last decade, the emergence of plane-wave ultrasound techniques, which can increase the imaging frame rate by two orders of magnitude, has greatly expanded the capability of medical ultrasound imaging. Furthermore, the advent of microbubble contrast agents can also significantly enhance the ultrasound signal from blood. Using high frame rate imaging techniques and microbubbles contrast agents, we have recently demonstrated the feasibility of the SDC method both *in vitro* and *in vivo*, showing that the maximum measurable through-plane flow velocity can be well over 1 m/s, which is physiologically equivalent to most flow in the cardiovascular system (Zhou et al. 2018). However, the current SDC method has an intrinsic limitation on differentiating the flow direction in the blood vessel. In other words, it can only estimate the through-plane flow speed but not the direction. In many parts of the cardiovascular system, there is bidirectional flow (reverse flow will occur) during certain periods of the cardiac cycle, especially when diseases exist in the vessel (Sutton et al., 1994, Bukachi et al., 2005, Tahmasebpour et al., 2005, Leow et al., 2015). Therefore, knowing the direction of the through-plane flow is crucial to accurately estimating the flow volume.

In this study, our aim is to detect the through-plane flow direction in the SDC method so that accurate estimation of the flow volume can be achieved even when the flow is bidirectional. The idea is to rotate the probe to have a tilted angle between the scanning plane and the vessel radius direction while implementing the conventional SDC method. In this way, the through-plane flow direction can be differentiated based on the in-plane flow direction by assuming that the blood flow primarily moves along the longitudinal direction. Feasibility of this method was investigated using computer simulations and experimental flow phantoms.

## Methods

### Theory in ultrasound SDC for estimating through-plane flow speed

**Fig. 1:**
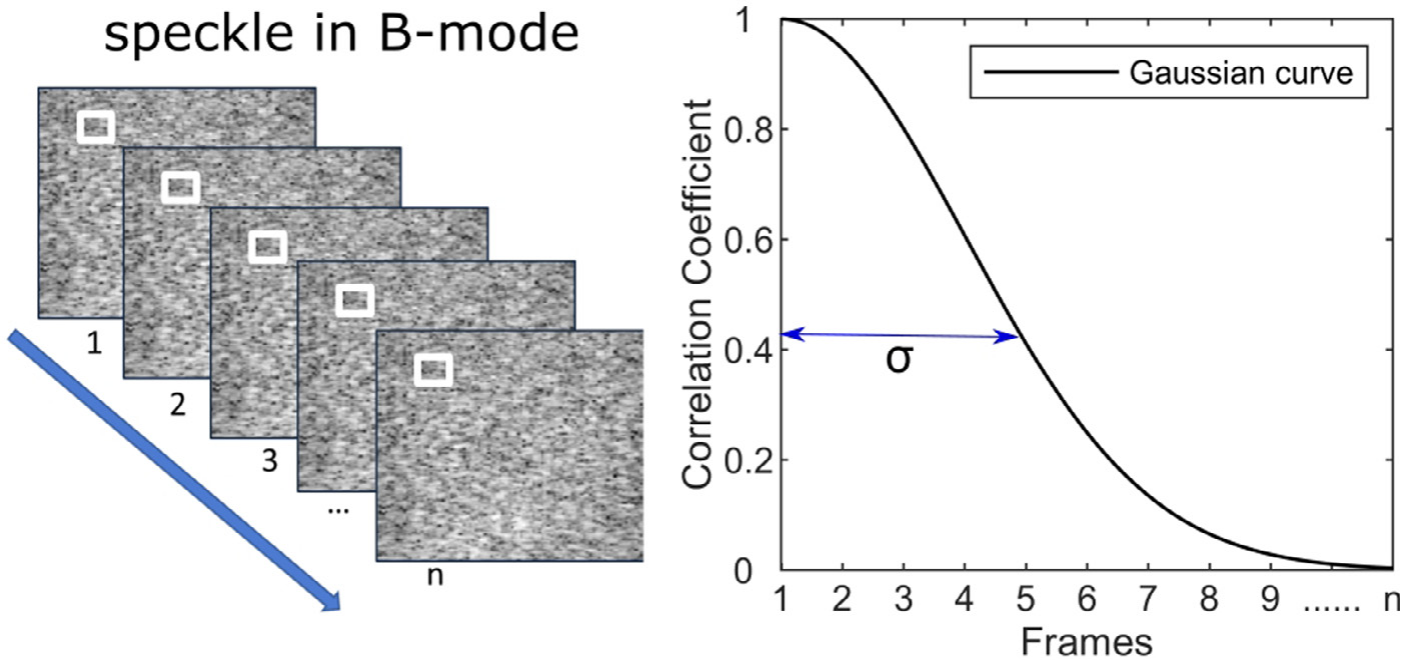
Intensity values between a series of patches (white boxes) from B-mode frames (frame 1 to frame n) decorrelate and the decorrelation rate follows a Gaussian curve.

Because of acoustic wave interference, ultrasound B-mode images are represented as speckle patterns (Burckhardt, 1978, Wagner et al., 1983). The spatial speckle patterns from two imaging frames could decorrelate when the relative position between scatterers and the probe changed. By assuming a Gaussian-shaped resolution cell, the relationship between the displacement and the decorrelation rate follows a zero-mean Gaussian curve as shown in eqn (1) and Figure 1.

(1) $$C\left(n\right)=\;e^{\frac{-{\left(n\cdot \Delta d\right)}^{2}}{2\sigma^{2}}}=\;e^{\frac{-{\left(n\cdot v\cdot \Delta t\right)}^{2}}{2\sigma^{2}}}=e^{\frac{-{\left(n\cdot \Delta t\cdot D\right)}^{2}}{2}},$$

where *C* is the correlation coefficient of the B-mode intensity values between the first frame (n=0) and the following frames, *n* is the lag number, Δ*d* is the displacement between two consecutive frames, Δ*t* is the reciprocal of the imaging frame rate, *v* is the scatterer velocity, *σ* is the overall beam correlation width (BCW) relating to dimensions of the resolution cell, and D=v/σ is known as the decorrelation value which is obtained from the B-mode image data through curve fitting. Because the imaging frame rate could be very high (up to 10 k fps), the velocity *v* was assumed to be constant in the consecutive frames used in curve fitting.

By assuming the ultrasound image formation is separable into factors arising from the three orthogonal directions in the space (Wagner et al. 1983), the overall decorrelation value *D* was then decomposed as the linear summation of decorrelation from three orthogonal directions (lateral, elevational and axial) (Rubin et al., 2001, Tuthill et al., 2002):

(2) $$D^{2}=\;\frac{{v_{x}}^{2}}{{\sigma_{x}}^{2}}+\;\frac{{v_{y}}^{2}}{{\sigma_{y}}^{2}}+\;\frac{{v_{z}}^{2}}{{\sigma_{z}}^{2}},$$

where *v_x_, v_y_* and *v_z_* are the scatterer velocity in the lateral direction, elevational direction and axial direction, respectively, *σ_x_, σ_y_* and *σ_z_* are the BCWs relating to the resolution cell in the corresponding directions. The decorrelation value D was obtained by curve fitting image intensity over time with a Gaussian curve as depicted in eqn (1). The values of *σ_x_, σ_y_* and *σ_z_* at each spatial position along the depth direction were calibrated using a fully developed speckle phantom (Tuthill et al., 2012, Zhou et al., 2018). In-plane velocities *v_x_* and *v_z_* were obtained using ultrasound imaging velocimetry (UIV) that tracks the speckle pattern in plane with a 2-D cross-correlation algorithm using the same B-mode data as in the curve fitting. In this way, the through-plane flow velocity *v_y_* was estimated using eqn (2).

### Detection of through-plane flow direction

**Fig. 2:**
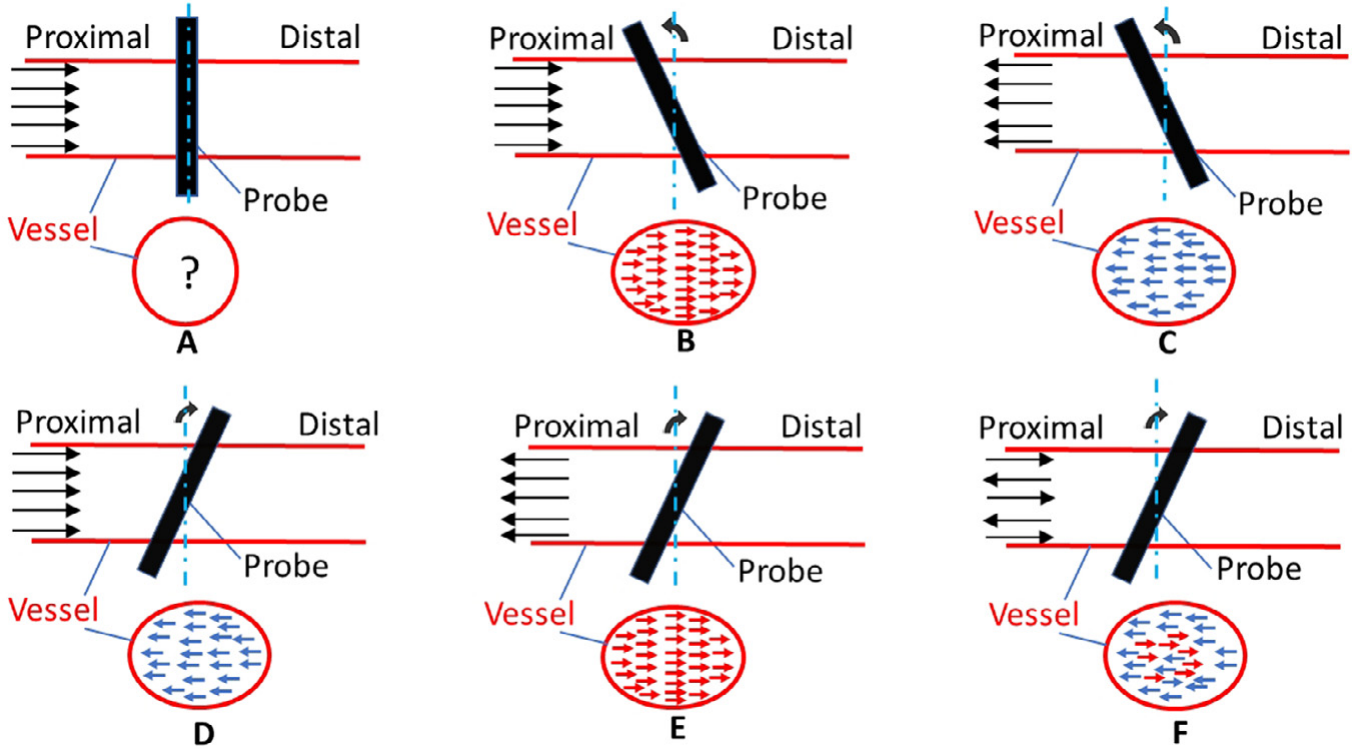
Illustration of the ultrasound scanning strategy in the proposed method.

In our previous SDC method (Zhou et al. 2018), the blood vessel was viewed in the transverse view (short axis view) as shown in Figure 2a. The upper part of the graph in Figure 2a illustrates the longitudinal view of the blood vessel being scanned by an ultrasound transducer which is represented by the rectangle positioned on top of the blood vessel at a 90° angle. The image of the blood vessel then is formed from the transverse view, which is a circle as shown in the lower part of Figure 2a. Our previous SDC method can estimate the speed of flow going through the scanning plane. However, it could not distinguish its direction, resulting in potential errors in the estimation of volumetric flow.

In this study, the transducer was rotated from the previous 90° position to have a tilted view (Fig. 2b–f). The ultrasound image of the vessel's cross section is not a circle any more but an ellipse, as shown in the lower graphs in Figure 2b–f. By assuming that the flow is primarily moving along the long axis of the vessel, the through-plane flow direction at each spatial point of the ellipse can be distinguished from its corresponding in-plane flow direction which was tracked by UIV. For example, when the probe is tilted anticlockwise as in Figure 2b and the blood flow is moving from the left (proximal to the heart) to the right (distal to the heart) as shown by the upper graph in Figure 2b, then in-plane flow will move from left to right as shown by the lower graph. With the same tilted position, when the flow is moving in the opposite direction, the in-plane flow also changes its direction (Fig. 2c). The transducer can also be rotated clockwise to establish the through-plane flow direction as shown in Figure 2 (d, f). To improve the accuracy of the estimation of the flow volume, results from both anticlockwise and clockwise tilting were averaged to give one estimation. In Figure 2f, the blood flow in the vessel is bidirectional. This could happen during the transitions when flow changes its direction in the vessel within the cardiac cycle. In this case, the flow could move in both directions within the scanning plane.

In the implementation of this method, no matter which direction (clockwise or anticlockwise) the transducer was tilted, the through-plane flow at a specific position was initially defined as positive if the corresponding in-plane flow moves to the right and negative if in-plane flow moves to the left. Based on the initial definition, the net flow volume, which is the blood flow going downstream from the heart (from proximal to distal), was calculated within one complete cardiac cycle. If the calculated net flow volume turned out to be negative, its absolute value still represents the volumetric flow amplitude and the initial assumption of flow direction would be inverted.

### Validation on simulations

Computational fluid dynamics (CFD) and Field II (Jensen and Svendsen, 1992, Jensen, 1996) were used to simulate the bidirectional pulsatile flow and the ultrasound imaging procedure, respectively, to validate the feasibility of the proposed method against the ground truth.

**Fig. 3:**
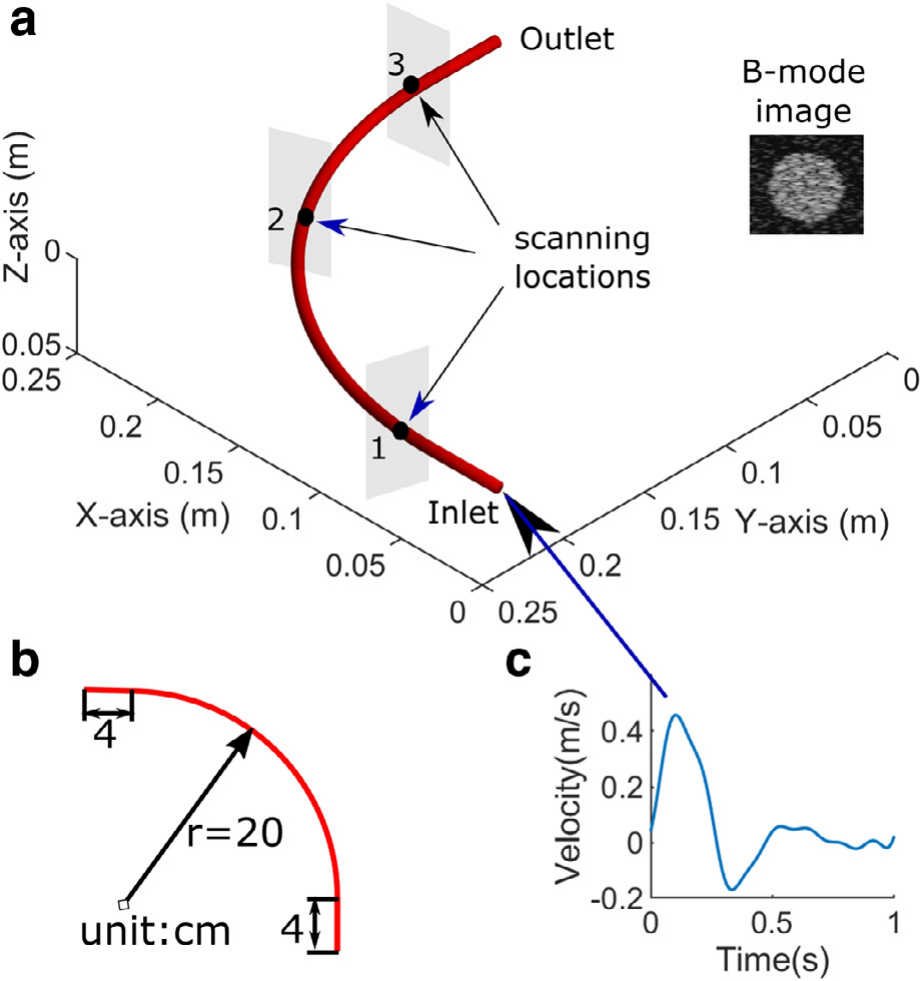
(a) The schematic illustration of CFD simulation of a curved vessel, the ultrasound scanning locations and view of the obtained B-mode image; (b) the dimensions of the vessel's centerline; and (c) the inlet mean velocity waveform. CFD = computational fluid dynamics.

**CFD simulation of bidirectional blood flow***.* To establish the through-plane flow direction, the in-plane flow direction needs to be detected. Through-plane flow direction can be perfectly established if all the flow moves parallel to the vessel's long axis, such as in a straight vessel. However, in the human body, the physiologic flow has secondary components when the blood vessel is not straight or when it is curved. For example, spiral flow along the aortic arch can be induced by the heart (Alex et al. 2012). To investigate the robustness of the proposed method, the vessel geometry simulated in the CFD has both straight and curved features (Fig. 3). Curved vessel generates secondary flow and a typical parameter to measure the scale of secondary flow is the dimensionless Dean number (Ku 1997).

(3) $$\begin{array}{c} Dean={\left(2\delta \right)}^{\frac{1}{2}}\cdot 4Re\\ \delta =\frac{r}{R}, \end{array}$$

where *r* is the vessel radius, *R* is the radius of the curvature of centreline and *Re* is the Reynolds number. The Dean number mapping in the cardiovascular system is not available in the literature, but it was found that a normal Dean number in the abdominal aorta is roughly about 260 (Ku 1997).

In this study, the inlet boundary condition in the CFD simulation was based off a typical flow pattern in a human's common femoral artery where reverse flow exists. The Fourier series coefficients (Table 1) of the mean flow velocity waveform *V_mean_*(*t*) estimated by Doppler method was adapted from published data (Evans et al. 2000). The mean velocity waveform in a straight tube can be represented by the Womersley equations. The peak Reynolds number was about 833, from which the maximum Dean number in the simulated flow was calculated from eqn (3) as being about 550. This is larger than the Dean number of 260 found in the abdominal aorta, because the secondary flow could be larger in other parts of the cardiovascular system, such as the heart or the ascending aorta. The outlet was chosen to have constant atmospheric pressure and non-slip rigid wall condition was applied in the CFD model. Blood was assumed to be a non-compressible Newtonian fluid having the typical properties of normal healthy human blood, a density of 1060 kg·m^−3^ and dynamic viscosity of 3.5 mPa · *s*. STAR-CCM+ (V11.06, Siemens, Berlin, Germany) was used as the solver for the Navier-Stokes equations to obtain the full-flow velocity field in the 3-D domain. The time step for results output was set to 10 ms; thus, 100 values were available for the velocity variable within one cardiac cycle (1 s). The grid size was determined by the grid convergence. The CFD simulation took 1 h to complete with a 64-bit, 3.40-GHz Intel Core i7-4770 processor.

**Table 1:** The parameters of the mean flow velocity for the Womersley equations

Diameter = 6 mm			
Heart rate = 60 bpm			
Blood viscosity = 0.0035 kg·m^−1^ s^−1^			
Blood density = 1060 kg·m^−3^			
$V_{mean}\left(t\right)=\;\sum_{n\;=\;0}^{n\;=\;8}V_{n}\cdot \text{cos}\left(2\pi f_{n}t+\varphi_{n}\right)$			
n	*f_n_* (Hz)	*V_n_* (m·s^−1^)	φ_*n*_ (degree)
0	0	0.066	0
1	1	0.126	32
2	2	0.166	85
3	3	0.085	156
4	4	0.021	193
5	5	0.018	133
6	6	0.021	155
7	7	0.019	195
8	8	0.0007	310

Note. *V_mean_*(*t*) is the mean flow velocity waveform in the artery.

**Ultrasound simulation with Field II***.* Based on the flow velocity field from CFD, simulated scatterers updated their positions within the 3-D vessel domain spatially and temporally, and the Field II was used to generate simulation images (Swillens et al. 2009). About 10 randomly located scatterers were defined in each resolution cell. High-frame-rate plane wave ultrasound was simulated to collect the B-mode images of the moving scatterers. The vessel was located at about 20 mm in the imaging depth. The parameters used in the Field II simulation are given in Table 2. Simulated ultrasound data were collected from three different locations: the straight part of the tube near the inlet (location 1), the middle of the curved part (location 2) and the straight part of the tube near the outlet (location 3) as shown in Figure 3. Secondary flow was expected at locations 2 and 3. Random Gaussian noise was added to the simulated ultrasound data to have a signal to noise ratio of 20 dB.

**Table 2:** Parameters in the Field II simulation

Parameter	Value
Central frequency	8 MHz
Excitation pulse	Sinusoid
Pulse cycles	2
Sound velocity	1540 m/s
Sampling frequency	100 MHz
Element width	170 µm
Element height	5 mm
Kerf	30 µm
Element number	128
Frame rate	8000 Hz
Imaging mode	Plane wave

### In vitro validation

**Fig. 4:**
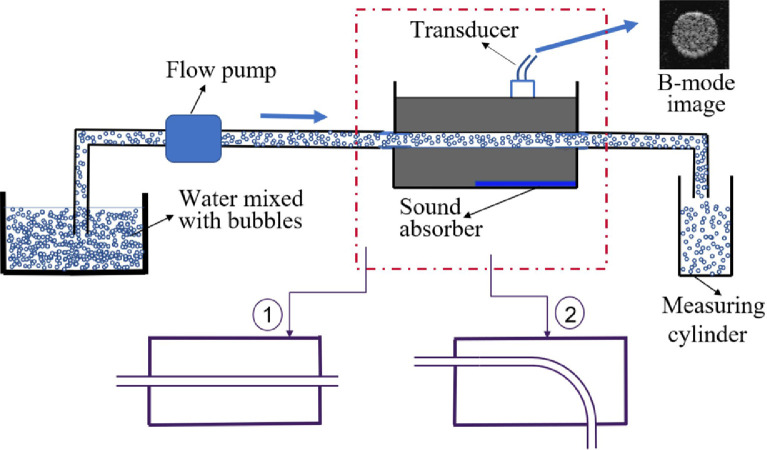
Diagram of the flow phantoms and the view of obtained B-mode images. Vessel in the red rectangle area could be a straight one or a curved one.

Both straight vessel and curved vessel were investigated in the experiments. The straight vessel phantom was an in-house designed polyvinyl alcohol-cryogel (PVA-c)-based wall-less phantom fabricated in a box with a luminal diameter of 5 ± 0.1 mm. The PVA-c phantom (15% PVA and 85% water by mass) was made from three freeze-thaw cycles of the PVA solution, reported to give tissue-like acoustic properties (speed of sound and attenuation) (King et al. 2011). The curved vessel phantom was made of a natural rubber tube (6 mm in diameter, WZ-06448-18, Cole-Parmer, UK) which was submerged in water and bent to have a similar curvature as in the CFD simulation, with a maximum Dean number of about 800. A piston pump (Harvard Apparatus, Kent, UK) was connected to the vessel phantom which generated a pulsatile flow (about 80 cycle/min) at a flow rate of about 75 mL/min. The working fluid was water mixed with decafluorobutane microbubbles as contrast agents. A diagram of the flow phantom setup is given in Figure 4 where the vessel (in the red rectangle area) being scanned by ultrasound could be straight or curved.

A transverse view of the vessel at the scanning location was obtained at first with a L12-3 v probe connected to a Vantage Veraonics 128 system (Kirkland, WA, USA). Then for collecting data, the probe was tilted anticlockwise manually by about 10^o^ along the depth direction (Fig. 2). Single angle plane wave imaging (no steering of the transmitting plane) with a frame rate of 8000 Hz was used. Two to three pulsatile cycles (about 80 cycle/min) of data were obtained each time. The same measurement was made to collect the data by tilting the probe clockwise. Each measurement was repeated three times. The transmitted sound wave had a central frequency of 8 MHz and a mechanical index (MI) of 0.19 (calibrated in water). A relatively low MI is necessary in this study to avoid significant microbubble destructions which could cause decorrelation and lead to overestimation of the flow velocity. All the experiments were conducted at room temperature (20 ± 1°C).

Separate measurements were made to scan the vessel at the same location but using the longitudinal view and applying the UIV method to get the velocity reference. Data collected by UIV technique were also repeated three times. The reference flow rate was obtained by directly measuring the fluid leaving the phantom with a measuring cylinder and a stopwatch.

### Data processing

Radio frequency data generated from simulation or acquired from *in vitro* experiment were beamformed into B-mode images (without log compression). Segmentation was then performed by manually selecting the lumen in the first frame and dynamically tracking the vessel wall in the subsequent frames using a localized region-based active contour segmentation (Lankton and Tannenbaum 2008). The decorrelation algorithm was applied to the segmented luminal area to estimate the 2-D through-plane velocity *v_y_* in eqn (2) as it was done in a previous study (Zhou et al. 2018). Specifically, in eqn (2), the BCWs *σ_x_, σ_y_* and *σ_z_* in Field II simulation were calibrated by scanning a simulated speckle phantom and in experiments by scanning a speckle phantom with the transducer fitted on a computer-controlled translation stage; the in-plane flow velocities (*v_x_* and *v_z_*) were tracked by the UIV method based on the B-mode images (without log compression). With all these variables available, the through-plane velocity *v_y_* was derived for each spatial position in the lumen from eqn (2). Finally, the volumetric flow rate was calculated by integrating velocity *v_y_* over the dynamically segmented luminal area.

## Results

### Simulations

**Fig. 5:**
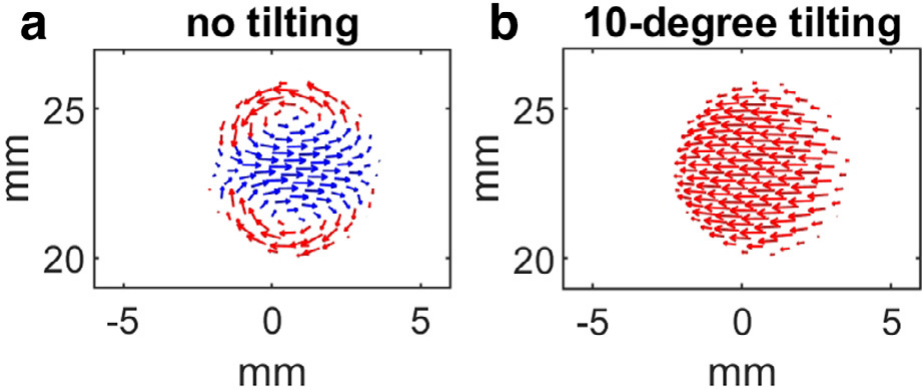
The in-plane flow patterns under the nontilted transverse view (a) and the 10° tilted transverse view (b) at the scanning location 2 in simulation. Flow direction and magnitude were color coded.

The peak systolic in-plane flow velocities at location 2 (Fig. 3) from the CFD simulation are given in Figure 5. It showed that in-plane flow direction can be used to detect through-plane flow direction with a tilted scanning view. A vortex can be seen in the non-tilted transverse view due to the curved geometry, and through-plane flow direction cannot be derived from the in-plane flow direction if the vessel was scanned under this view. When the scanning plane was tilted by 10 degrees, in-plane velocities throughout the vessel lumen turned out to have the same direction when the flow is going forward at peak systole. A movie showing the in-plane flow patterns under these two views throughout the cardiac cycle is given in the Video S1.

**Fig. 6:**
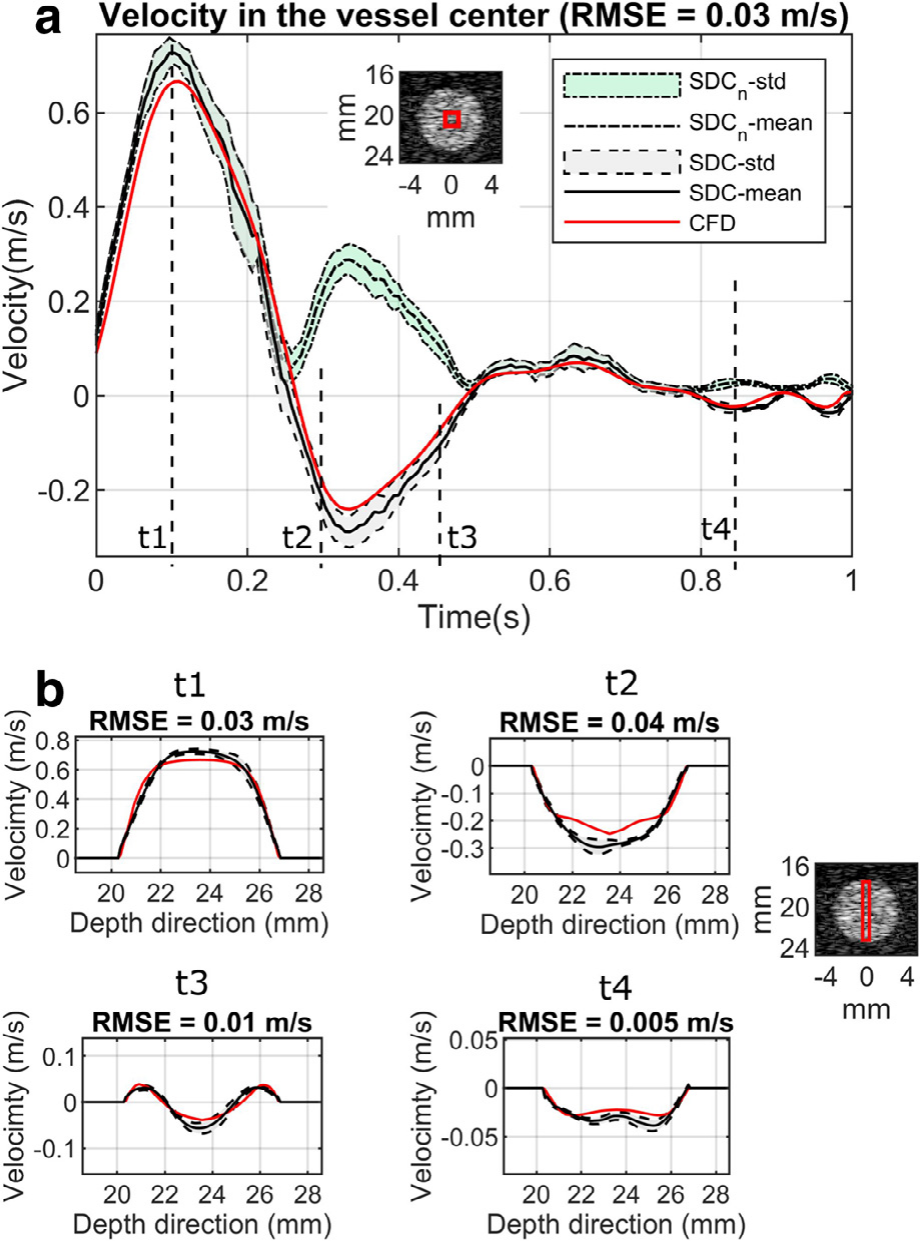
Comparisons of velocity between SDC method and CFD reference over a cardiac cycle at the scanning location 3. (a) Velocity from a 1-mm square area in the vessel center (indicated by the red box in the B-mode image), compared with the CFD reference. SDC_n_-std stands for the velocity standard deviations within the red box in the conventional SDC method where no direction detection was applied, and SDC_n_-mean stands for the corresponding mean values; SDC-std stands for the velocity standard deviations within the red box in the proposed SDC method, and SDC-mean stands for the corresponding mean values. (b) Velocity profiles along the vessel's short axis within 1-mm width rectangle (indicated by the red box in the B-mode image on the right) at four different time points t1, t2, t3 and t4 indicated in (a), compared with the CFD reference. Curves in (b) share the legends with (a). SD = speckle decorrelation; CFD = computational fluid dynamics; RMSE = root mean square error.

The through-plane flow velocities at three different scanning locations can be accurately estimated using the proposed SDC method. The results from location 3 (Fig. 3), where the largest secondary flow is expected, are given in Figure 6, together with the reference from the CFD flow field. The estimated and reference flow velocities within a 1-mm square area in the center of the vessel were compared throughout the cardiac cycle. A comparison of the velocity profile across the vessel's short axis was also made at four different cardiac phases indicated by the dashed line in the Figure 6a. It demonstrated that the through-plane flow direction can be obtained by the proposed method. A movie illustrating the 3-D velocity profile in the scanning plane at location 2 over one cardiac cycle is provided in Video S2.

**Fig. 7:**
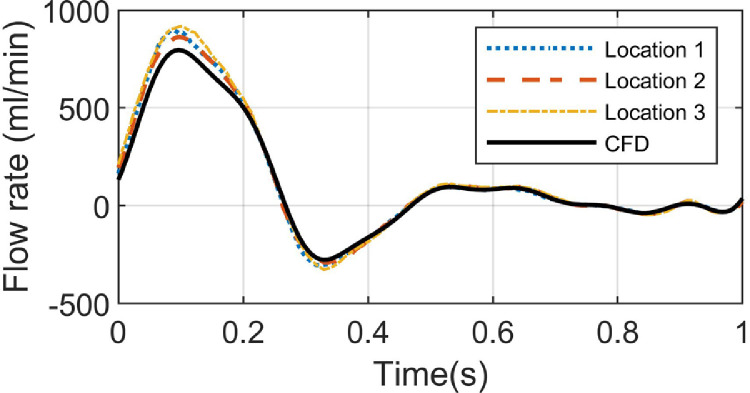
Comparisons of flow volume waveform at three different locations with the CFD reference volume waveform. CFD = computational fluid dynamics.

The temporal flow rates were also evaluated against the CFD ground truth and the results are shown in Figure 7. The true mean flow rate within one cardiac cycle was 121.2 mL/min, and the estimated mean flow rates at three scanning locations were 133.2, 134.0 and 141.6 mL/min, respectively. However, the estimated mean flow rates were 238.4, 256.5 and 265.3 mL/min if the proposed direction detection method was not applied. In these simulations, both flow velocity and rate tended to be slightly overestimated by the proposed SDC method compared with their references and explanation on this will be given in the discussion.

### *In vitro* experiment

**Fig. 8:**
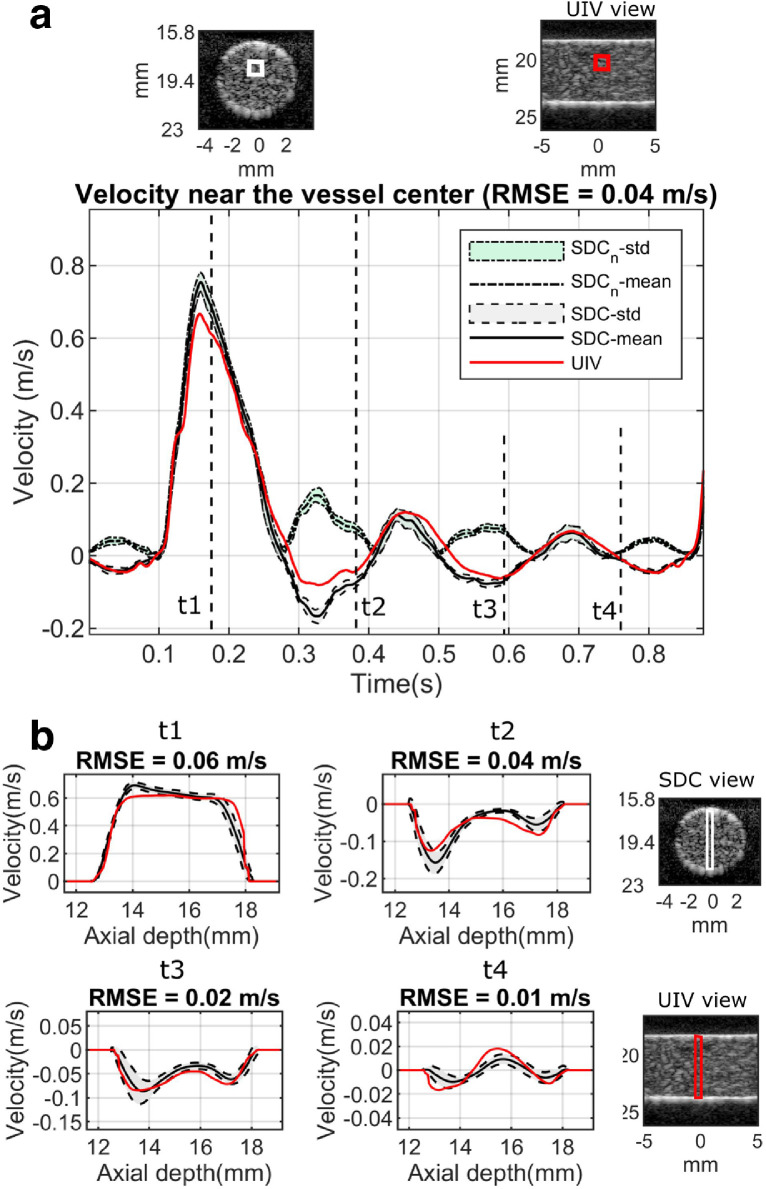
Comparisons of velocity between SDC method and UIV reference in the straight tube. (a) Averaged velocity from a 1-mm square area located at 1 mm away from the vessel center (indicated by the white box in the B-mode image), compared with the corresponding UIV reference (indicated by the red box). SDC_n_-std stands for the velocity standard deviations from six repeated measurements in the conventional SDC method where no direction detection was applied, and SDC_n_-mean stands for the corresponding mean values; SDC-std stands for the velocity standard deviations from six repeated measurements in the proposed SDC method, and SDC-mean stands for the corresponding mean values. (b) Velocity profiles along the vessel's short axis within 1-mm width rectangle (indicated by the white box in the B-mode image on the right) at four different time points t1, t2, t3 and t4 in one cardiac cycle, compared with the UIV reference (indicated by the red box). Curves in (b) share the legends with (a). SDC = speckle decorrelation; UIV = ultrasound imaging velocimetry; RMSE = root mean square error.

Comparisons of the estimated flow velocity between the SDC method and the UIV method in a straight vessel phantom are illustrated in Figure 8. It shows that flow velocity estimated from both methods matched well. A movie showing that in-plane flow in the lateral direction changed its directions when the through-plane flow changed direction is given in Video S3. The volumetric flow rate calculated from the proposed SDC method was 71.2 ± 3.0 mL/min while the reference flow rate from the timed collection was 75.6 mL/min, indicating only a 5.8% underestimation. The estimated flow rate was 155.8 ± 5.8 mL/min when no direction detection was applied, which is a 106.0% overestimation.

**Fig. 9:**
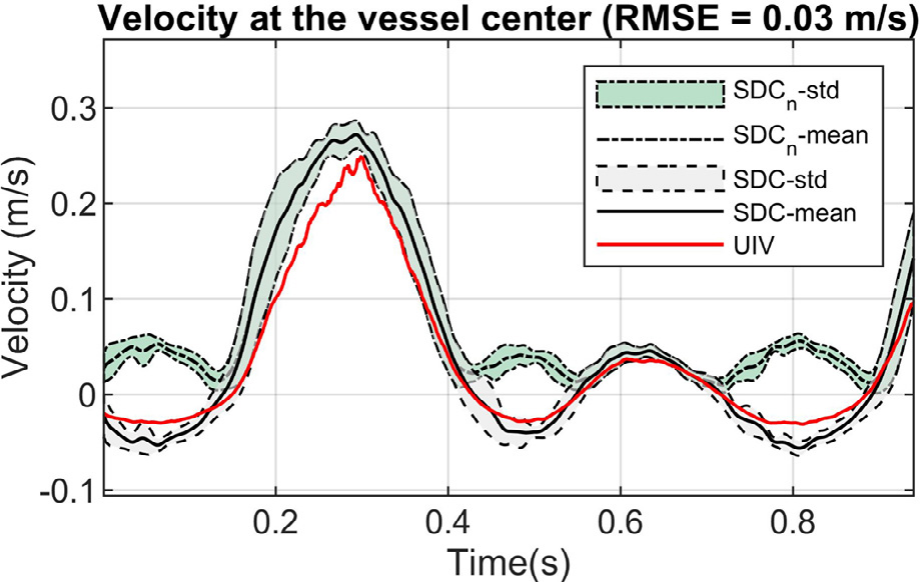
Comparisons of velocity between SDC method and UIV reference in the curved tube. Explanations on those curves can be found in Figure 8a. SDC = speckle decorrelation; UIV = ultrasound imaging velocimetry.

**Fig. 10:**
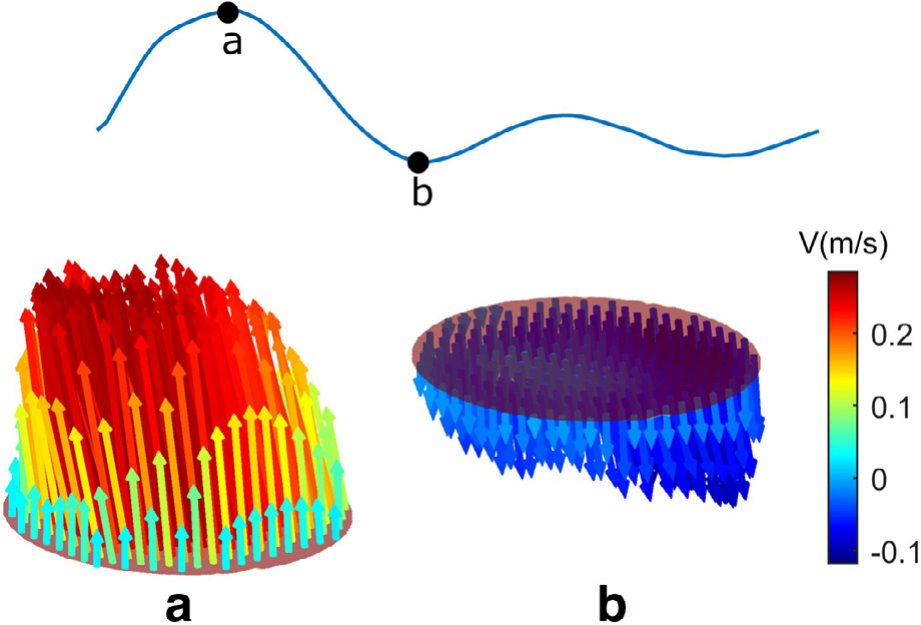
The 3-D velocity profile from the curved vessel at two temporal positions: at peak forward flow (a) and at peak backward flow (b).

In the curved vessel, the SDC method can also distinguish the through-plane flow direction compared with the UIV reference (Fig. 9). Only the flow velocity from the central area of the vessel was investigated in this case because it was difficult to get a reliable velocity reference across the vessel's short axis from the longitudinal view in the UIV method when the vessel was curved. The 3-D velocity profile obtained from the proposed method in the curved vessel is illustrated at two temporal points (with peak forward and peak backward flow) in the cardiac cycle (Fig. 10). A movie showing the corresponding 4-D velocity profile is given in Video S4. The reference flow rate from the timed-collection method was 75.0 mL/min and the estimated flow rate by the proposed SDC method was 69.6 ± 4.9 mL/min, with a 7.2% underestimation. The flow rate was significantly overestimated (157.5 ± 5.9 mL/min, 110.0% overestimation) when no direction detection method was applied.

## Discussion

Ultrasound SDC provided a unique way to estimate volumetric flow in the blood vessel by imaging the vessel in the transverse view and estimating the spatially and temporally resolved through-plane flow velocity. In this study, a key limitation of the current SDC method, the lack of differentiation of flow direction which is important for volumetric estimation of some physiologic flow, was addressed by a newly proposed method through tilting the transducer from the transverse view and processing the in-plane flow information.

To our knowledge, it is the first time for a study to directly measure the volumetric flow from its 2-D velocity profile in the presence of reverse flow, by a 1-D array probe without the need to assume the flow profile in the vessel or to obtain the knowledge of the beam angle relative to the vessel. Most other ultrasound techniques using a 1-D array transducer must assume an analytical or symmetric velocity profile, based on which the flow volume is estimated either from the velocity measurements at a point by spectral Doppler (Zhou et al. 2016) or from 2-D velocities by vector flow techniques in the longitudinal scanning plane (Jensen and Munk, 1998, Yiu and Yu, 2016, Leow and Tang, 2018). The proposed ultrasound method in this study has overcome those limitations with a conventional 1-D array ultrasound transducer, which makes it easy to implement. The improvement in accurately estimating the flow volume even in the presence of reverse flow makes the proposed method a promising technique in a range of clinical applications as mentioned in the introduction. In addition, this method might be more informative than the spectral Doppler in detecting a total occlusion by providing a 2-D cross-sectional velocity profile, but this requires further *in vitro* and *in vivo* validations.

The feasibility of this method was demonstrated on straight and curved vessels by simulations and flow phantoms. It can estimate through-plane flow velocity with good accuracies in terms of magnitude and direction, which is not possible in previous decorrelation studies (Rubin et al., 2001, Zhou et al., 2018). It should be noted that all the negative parts in those SDC-estimated curves in Fig. 6, Fig. 8 and 9 were mistaken as positive if the proposed method were not applied, leading to more than 100% overestimation in volumetric flow rate in the demonstrated scenarios. Therefore, a significant improvement was achieved by the proposed method with the ability of detecting through-plane flow direction. Currently, there is no other existing solution for this problem in terms of differentiating the blood flow direction in the ultrasound SDC method.

Some overestimations of through-plane flow velocity can be seen in each case. These overestimations have been shown to be caused by the SDC method which tends to overestimate the through-plane flow velocity if the in-plane flow velocity is large (Rubin et al., 2001, Zhou et al., 2018). This error could be suppressed in traditional SDC method by having an absolute transverse view where the in-plane flow is small. In this study, more in-plane flow is introduced due to the tilted view which is required to detect the through-plane flow direction. To minimize this overestimation, the tilted angle should be kept small. In this work, a 10-degree angle was used.

Overestimation of flow velocity leads to overestimation of volumetric flow rate as expected in simulation. In flow phantom, the volumetric flow rate was slightly underestimated and despite the flow velocity was overestimated. This was because the detection of through-plane flow direction depends on the UIV-estimated in-plane flow direction. The UIV technique is not sensitive enough to detect the change of in-plane flow direction at some places where the flow is slow. This could lead to overall underestimation of flow rate since the positive flow always outweighs the negative flow from the physiologic view, which means more positive flow could be mistaken as negative flow within a cardiac cycle. Further studies to correct the overestimation should be explored. In addition, a natural rubber tube was used in the flow phantom for collecting data in the case of a curved vessel. Its acoustic properties were not calibrated in this study. The deviation of its acoustic properties from human tissue might also affect the flow estimation, but its effect should not be significant.

In this study, it was assumed that the blood flow in the vessel primarily moves along the vessel so that the through-plane flow direction can be worked out by tilting the scanning plane. Obviously, this method would work best at a condition where the blood vessel is straight. In human body, most blood vessels are not straight but curved to some degree. Secondary flow occurs with curved vessel, meaning that flow will not perfectly move along the vessel. The Dean number was adopted to measure the scale of secondary flow, and a Dean number of 260 was reported in the abdominal aorta. The Dean number would be larger when it comes to the flow in the heart, or the ascending aorta, so we chose to test this method on flows with larger Dean numbers which were maximally about 500 in simulation and 800 in flow phantom. The proposed method was demonstrated to be able to detect the through-plane flow direction despite the significant secondary flow. In the human body blood vessel geometries, such as bifurcations and branches, could be more complicated, leading to more complex blood flows or even turbulent flows. In these cases, titling the transducer might not be enough to effectively detect the flow direction. However, the transducer can be moved to scan an area where the flow is not turbulent or simpler because the flow volume will be conserved in a vessel.

Microbubbles, which have been widely used in clinical practice and research (Tang et al. 2011), were used in the flow phantom as a contrast agent to obtain enhanced acoustic signals from the flow. The advantages of using microbubble for enhancing the signal-to-noise ratio (SNR) in the decorrelation analysis have already been shown in previous studies (Rubin et al., 1999, Zhou et al., 2018). In principle, the SDC method could work with native blood but extra clutter filtering might be required to improve the SNR in this case. This requires further study. Further *in vivo* validations of this method are also necessary.

## Conclusion

A new method was proposed and evaluated for detecting the through-plane flow direction in the SDC method, which would enable the decorrelation method to estimate the volumetric flow rate more accurately in the presence of flow reversal. This method is capable of accurately measuring the physiologic flow using a conventional 1-D ultrasound array probe, which could impact a wide range of its clinical applications.

## Appendix Supplementary materials

Image, video 1

Image, video 2

Image, video 3

Image, video 4
